# Antitussive Activity of Pseudostellaria heterophylla (Miq.) Pax Extracts and Improvement in Lung Function via Adjustment of Multi-Cytokine Levels

**DOI:** 10.3390/molecules16043360

**Published:** 2011-04-19

**Authors:** Wensheng Pang, Siding Lin, Qiwen Dai, Hongcheng Zhang, Juan Hu

**Affiliations:** School of Pharmacy, Fujian University of Traditional Chinese Medicine, Fuzhou, 350108, China; E-Mails: pws@fjtcm.edu.cn (W.P.); linsd1985@126.com (S.L.); dqw3718150@163.com (Q.D.); arken20@163.com (H.Z.)

**Keywords:** *Pseudostellaria heterophylla* (Miq.) Pax, extracts, antitussive activity, improvement lung function, multi-cytokine adjusting

## Abstract

*Pseudostellaria heterophylla* (Miq.) Pax is one of the most widespread herbal and healthcare products in China. Extensive clinical use has shown that it has functions which “strengthens qi and generates saliva, moistens the lung and relieves cough”. The ethyl acetate fraction extracted from the roots of the plant *Pseudostellaria heterophylla* exhibited a dose-dependent antitussive effect between 100 to 500 mg/kg. At a dose of 400 mg/kg, the ethyl acetate fraction treatment markedly prolonged the cough latent period and reduced the number of coughs in a guinea pig model induced by citric acid. Fall lung airway resistance, rise in dynamic lung compliance, decreased serum levels of IL-8, GM-CSF, TNF-α, and ET-1 in rat model of stable phase chronic obstructive pulmonary disease induced by cigarette smoke exposure were also observed. These results suggest that ethyl acetate fraction has antitussive activity related to its improvement in lung function via attenuation of airway inflammation by adjustment of multi-cytokine levels.

## Abbreviations

TZS: *Pseudostellaria heterophylla* (Miq.) Pax, Taizishen
COPD: chronic obstructive pulmonary disease
MedLab: bio-signal acquisition and processing system
EtOHE: ethanol crude extract
WE: water extract
EtOAcF: ethyl acetate fraction
*n*-BuOHF: *n*-butanol fraction
PEF: petroleum ether fraction
R_L_: lung airway resistance
C_dyn_: dynamic lung compliance
*Ptp*: trans-pulmonary pressure
V: rate of air flow
ELISA: enzyme linked immunosorbent assay
CS: cigarette Smoking
Kechuanning capsules: Golong brand Kechuanning capsules
GM-CSF: granulocyte macrophage-colony stimulating factor

## 1. Introduction

Cough is an indispensable defensive reflex and is also a common symptom of diseases such as asthma, chronic obstructive pulmonary disease (COPD) and lung cancer. Control of cough remains a major unmet medical need and, although the centrally acting opioids have remained the antitussive drug of choice for decades, they possess many unwanted side-effects such as sedation and gastrointestinal symptoms [1]. 

In Chinese clinical medicine, the study of lung disease focuses the syndrome of deficiency on the lung that is usually accompanied by stagnation of phlegm and blood. Many plants have been utilized for thousands of years to treat respiratory complaints such as cough, asthma, bronchial affections, pneumonia and expectoration [2,3,4,5]. 

As described in the Pharmacopoeia of People’s Republic of China [6], *Pseudostellaria heterophylla* (Miq.) Pax (Taizishen, TZS) has been used as a drug for the treatment of various diseases associated with lung asthenia, dry cough, as well as a spleen tonic. Recent research indicates that TZS contains antifungal, antifatigue, immunologic enhancement and antioxidant properties [7,8,9]. TZS is a mild medicine to tone up the body and a good ginseng substitute [10,11]. 

Based on the uses of this plant in traditional medicine, the present study was undertaken to evaluate the antitussive activity of aqueous, ethanol, petroleum ether, ethyl acetate and *n*-butyl alcohol extracts of TZS against citric acid-induced cough reflex in guinea pigs.

## 2. Results and Discussion

### 2.1. Antitussive potency of TZS extracts

**Figure 1:**
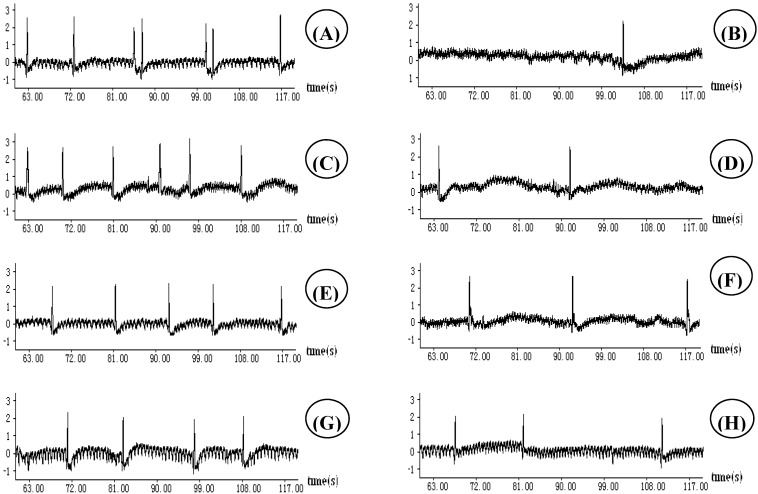
Effect of extracts of *Pseudostellaria heterophylla* at the dose 400 mg/kg on respiratory waves of guinea pigs recorded by a pressure transducer connected to a biology signal collection processing system: (A) citric acid induced. (B) codeine phosphate + citric acid. (C) PEF + citric acid. (D) EtOAcF + citric acid. (E) *n-*BOHF + citric acid. (F) EtOHE + citric acid. (G) WE + citric acid; (H) Guilong Kechuanning + citric acid.

A citric acid solution was used to induce cough in guinea pigs in order to screen the antitussive activities of TZS. The respiratory wave patterns were recorded by a pressure transducer connected to the MedLab instrument as shown in **Figure 1.** The ethyl acetate fraction (EtOAcF) significantly lowered the cough episodes at doses of 200 and 400 mg/kg intragastric administration (i.g.). Ethanol crude extract (EtOHE) only showed significant antitussive effects at 400 mg/kg i.g. The animals given *n*-butanol fraction (n-BuOHF) at 400 mg/kg i.g. had sluggish or unsteady movements. These side effects were not observed in animals given equivalent doses of water extract (WE), EtOHE, petroleum ether fraction (PEF) or EtOAcF by the same route of administration.

Using a seven minute period for calculation purposes, cough episodes during the first challenge were recorded as control data (*Cc*) and numbers of coughs produced after test drug administration as *Ct*. The antitussive effect was expressed as the % inhibition of cough = [(*Cc*-*Ct*)/*Cc*] × 100. 

**Figure 2:**
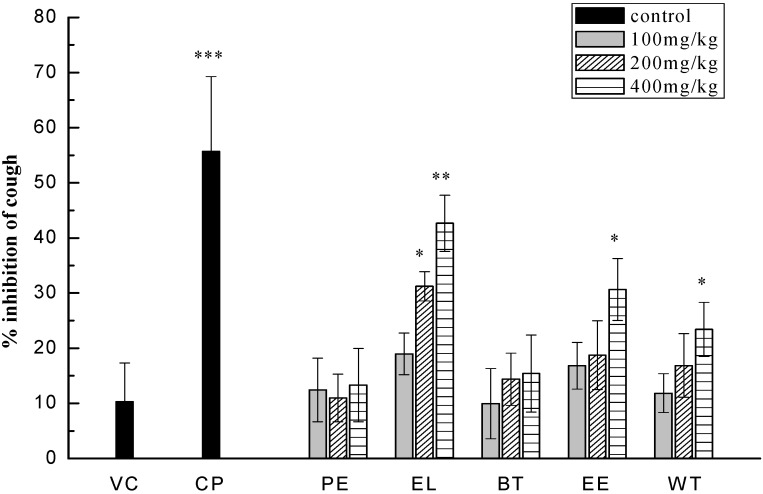
Cough inhibition of vehicle control (VC), positive codeine phosphate (CP), PEF (PE), EtOAcF (EL), *n*-BuOHF (BT), EtOHE (EE), WE (WT) at 100 mg/kg, 200 mg/kg, 400 mg/kg (i.g). **P < 0.01, *P < 0.05, n = 6.

**Figure 3:**
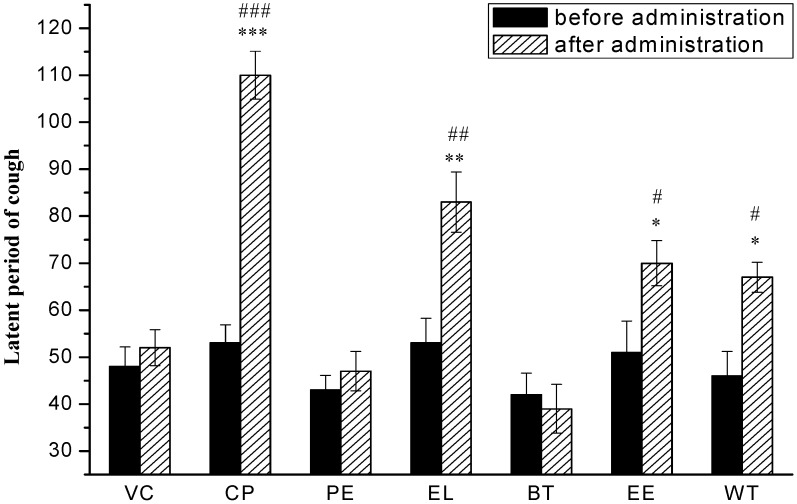
Latent period of cough of VC, positive codeine phosphate (CP), PEF (PE), EtOAcF (EL), *n*-BuOHF (BT), EtOHE (EE), WE (WT) at 400 mg/kg (i.g). * p < 0.05, ** p < 0.01, compared with vehicle control after administration; ^#^ p < 0.05, ^##^ p < 0.01, compared with data before administration, n = 6.

Statistical analysis of the data confirmed the difference in cough inhibition between groups treated by the five extracts at 100 mg/kg, 200 mg/kg, and 400 mg/kg i.g. At the high dose of 400 mg/kg, pharmacological model comparisons using the T-test confirmed a significant difference in cough inhibition for EtOAcF (42.65% inhibition, p < 0.01), EtOHE (30.62% inhibition, p < 0.05), WE (23.41% inhibition, p < 0.05). PEF and n-BuOHF treatments did not show significant differences compared to the vehicle (**Figure 2)**. The effects of different extracts of TZS at the dose of 400 mg/kg on magnifying the latent period of cough in guinea pigs are shown in **Figure 3**. There were significant differences in the effects of the positive control, codeine phosphate, and EtOAcF, EtOHE, and WE compared with the vehicle. The antitussive effect of EtOAcF was the highest among the test groups at a single dose of 400 mg/kg for 7 days about 90 min after administration. 

### 2.2. EtOAcF improves pulmonary function

Lung airway resistance (RL) is the opposition to flow caused by the forces of friction. It is defined as the ratio of trans-pulmonary pressure (Ptp) to the rate of air flow (V), RL = Ptp/V. Resistance to flow in the airways depends on whether the flow is laminar or turbulent, on the dimensions of the airway, and on the viscosity of the gas. Airway resistance decreases as lung volume increases because the airways distend as the lungs inflate, and wider airways have lower resistance. Lung compliance means the per unit change in pulmonary pressure caused by lung volume changes. It can be divided into dynamic compliance and static compliance. Lung tissue in the respiratory movement during movement of the respiratory muscles adapt to the extent shown as dynamic lung compliance, compliance, better, more unobstructed breathing and vice versa is not smooth, silicosis, emphysema and other diseases. Dynamic lung compliance (Cdyn) is defined as the ratio of tidal volume (Vt) to the rate of trans-pulmonary pressure, RL = Vt/ Ptp [16]. **Table 1** shows CS-induced changes of *R_L_* and *Cdvn* in stable phase COPD rats. In the model of CS-induced rats a 43.26% increase of *R_L_* and 64.45% reduction of *C_dyn_* with maximal response at 5 min compared to the baseline control were observed. After treatment with EtOAcF at 400 mg/kg i.g. for 15 days inhibited the action of cigarette on lung injury, the maximal increase of *R_L_* and reduction response of *C_dvn_* at 5 min was 7.09% ± 0.78% and 41.43% ± 9.35%, respectively. Compared to the stable phase COPD model, maximal reduction of *R_L_* and increased response of *C_dvn_* were 25.25% ± 7.67% and 64.76% ± 11.88%. Thus there was significant improvement in pulmonary function.

**Table 1:** Effect of EtOAcF on lung resistance and dynamic lung compliance challenged with cigarette inhalation in stable phase COPD rats.

Groups	V (mL/s)	Vt (mL)	Ptp (cm·H_2_O)	R_L_ (cm·H_2_O·s·mL^−1^)	C_dyn_ (mL·cm^−1^·H_2_O^−1^)
Control	657.80	34.69	18.55	0.0282 ± 0.0057	1.8702 ± 0.4588
Model	406.85	10.92	16.43	0.0404 ± 0.0048^▲▲^	0.6648 ± 0.2694 ^▲▲^
Kechuanning capsules	618.39	20.66	18.49	0.0299 ± 0.0065*	1.1174 ± 0.2794 *
EtOAcF	611.92	19.92	18.48	0.0302 ± 0.0079*	1.0953 ± 0.1904*

Values are expressed as mean ± S.E.M., n = 10; ^▲^ P ≤ 0.05 versus normal control, ^▲▲^ P ≤ 0.01 versus normal control; * P ≤ 0.05 versus sCOPD model, ** P ≤ 0.01 versus sCOPD model; ^C^ versus sCOPD model is NOT different

### 2.3. Changes in airway inflammatory cytokines in rat serum

Rat IL-8, IL-10, TNF-α, GM-CSF and ET-1 levels were quantified in serum using ELISAs specific for each cytokine, as shown in **Table 2**. The serum from the CS group exhibited markedly increased cytokines including neutrophil chemokines and inflammatory cytokines such as IL-8, TNF-α [17,18]. At a dose of 400 mg/kg EtOAcF, the release of the pro-inflammatory cytokines IL-8, TNF-α, GM-CSF, and ET-1 was significantly inhibited. The ELISA results showed that the anti-inflammatory cytokine IL-10 did not significantly increase after EtOAcF intervention.

**Table 2:** Changes of cytokine in rat serum with cigarette inhalation in stable COPD phase (ng/L).

Cytokine	Control	Model	Positive	EtOAcF
IL-8	598.41 ± 8.38	698.21 ± 22.96^▲▲^	614.76 ± 13.19*	604.16 ± 7.66 **
IL-10	29.20 ± 0.72	35.81 ± 1.88^▲▲^	56.90 ± 2.82**	36.59 ± 1.55^C^
TNF-α	93.25 ± 6.28	163.04 ± 16.39^▲▲^	93.96 ± 5.52**	95.37 ± 3.95**
GM-CSF	556.94 ± 22.07	722.87 ± 21.59^▲▲^	555.69 ± 21.32**	635.00 ± 19.70**
ET-1	171.55 ± 8.29	224.36 ± 8.72^▲▲^	179.17 ± 4.04**	211.53 ± 6.93 ^*^
IL-10/ TNF-α	0.31	0.219	0.610	0.384

Values are expressed as mean ± S.E.M., n = 10; ^▲^ P ≤ 0.05 versus normal control, ^▲▲^ P ≤ 0.01 versus normal control; * P ≤ 0.05 versus sCOPD model, ** P ≤ 0.01 versus sCOPD model; ^C^ versus sCOPD model is not significantly different.

COPD is a major global health problem with an increasing incidence and mortality [19,20]. COPD, currently believed to be an exaggerated inflammatory response to inhaled irritants, in particular cigarette smoke, causes progressive airflow limitation. This inflammation, where macrophages and neutrophils are prominent, leads to oxidative stress, emphysema, and small airway fibrosis and mucus hypersecretion. COPD responds poorly to current anti-inflammatory treatments, including potent glucocorticosteroids, and there is no specific drug treatment for this disease. 

Interleukin-8 (IL-8), one of the mediators which play a role in the pathogenesis of COPD along with LTB4 and TNF-α, is a cytokine with potent neutrophil chemotactic and activation properties [21]. ET-1 also has potent bronchoconstrictive properties, and has been implicated in the pathogenesis of airway disease. The role of ET-1 in the pathophysiology of COPD remains uncertain and may be related to its effect upon bronchoconstriction or its pro-inflammatory properties. Plasma ET-1 levels are elevated in COPD patients compared with control subjects [22]. TZS able to improve the lung function in rat serum with cigarette inhalation in stable COPD phase via adjustment of multi-cytokine Levels.

## 3. Experimental

### 3.1. Plant material

The roots of TZS were collectd in Zherong County, Fujian Province, China in July 2009. The plant was identified by Dr. M. Jin and a voucher specimen (No. 2010131037S) is deposited at the Fujian Pprovincial Institute for Drug Control, Fuzhou City, Fujian Province, China. The plant material was dried in a ventilated oven at 40 °C for 48 h and ground in a knife mill.

### 3.2. Instrument and reagents

MedLab_Model U/4c501H bio-signal acquisition and processing system (MedLab), with Model YP200 pressure transducer and Model HX200 pneumotachograph transducer was made by Nanjing Medease Science and Technology Co., Ltd. (Nanjing, China). Model DG5033A ELISA reader was made by Nanjing Medical Equipment Co., Ltd. (Nanjing, China). Model WH801 digital ultrasonic atomizer was purchased from Guangdong Yuehua Medical Equipments Co., Ltd. (Guangzhou, China). Codeine phosphate was obtained from Fujian University of Traditional Chinese Medicine Second Affiliated Clinical Hospital (Qinghai Pharmaceutical Factory Co. Ltd, batch No. 20091212). ELISA kits for IL-8, IL-10, ET-1, GM-CSF and TNF-α assay (R&D Systems, Inc., USA) were purchased from the Jiancheng Institute of Biotechnology. Ultrapure water was prepared using a Milli-Q system (Millipore, MA, USA). All other reagents and solvents were of analytical grade.

### 3.3. Extraction

The dried powder of TZS (20 kg) was extracted three times with 85% aqueous ethyl alcohol under reflux for 2 h,. After filtration and combination of the filtrates, they were concentrated to dryness in a rotary evaporator to provide an ethanol crude extract (EtOHE, 4,250 g). The EtOHE was re-dissolved in water and partitioned successively with three organic solvents to provide a petroleum ether fraction (PEF, 256 g), ethyl acetate fraction (EtOAcF, 84 g), and *n*-butanol fraction (n-BuOHF, 1,662 g). One kg of dried powdered plant was mixed with 5,000 mL of water under reflux for 2 h. The obtained water extract (WE) was expressed as the weight percentage of the total weight of the plant material, 13.69% (137 g). All the fractions were subjected to bioactive evaluation using the citric acid-induced guinea pig cough model.

### 3.4. Animals and grouping

All animals were purchased from Shanghai Slac Laboratory Animal Co., Ltd. (China) and kept at 20-25 °C and constant humidity 45-65% under a 12 h light-dark cycle with free access to food and water. The animal studies were approved by the Fujian Institute of Traditional Chinese Medicine Animal Ethics Committee (Fuzhou, China). The experimental procedures were carried out in accordance with the Guidelines for Animal Experimentation of Fujian University of Traditional Chinese Medicine (Fuzhou, China).

One hundred and two (102) qualified guinea pigs, body weight 220-250 g (skxk [Shanghai] 2009-0011) were randomly assigned to seventeen groups. Each group includes six animals, three from either gender. Guinea pigs were given drug intragastrically once a day for 7 days. The cough was induced by citric acid in guinea pigs at 45 min from the last administration. Control group received in a single oral dose the same volume of sterilized physiological sodium chloride solution that served as the vehicle control. Drug groups received extract (EtOHE, WE, PEF, EtOAcF, *n*-BuOHF) in a single intragastrically administered at high, medium and low (100 mg/kg, 200 mg/kg, 400 mg/kg). doses. For the positive group, codeine phosphate was given orally (10 mg/kg).

Forty (40) SD half male rats at SPF level, body weight 200-220 g (skxk[Shanghai] 2010-0018) were randomly assigned to four experimental groups. CS model group [12], a commercially available non-filter cigarette was used (Daqianmen brand cigarettes; Shanghai Tobacco Corporation, China). Each cigarette contained 0.9 mg of nicotine and 12 mg of tar. Animals were placed into a 1 m^3^ airtight chamber and exposed to the smoke equivalent of 10 cigarettes and 30 g sawdust for 30 min, one time each day, for 60 consecutive days. Control group animals were placed into the same type of apparatus as described in CS model group, and exposed to fresh air instead of CS. They were used as the fresh air control. CS model group and control group were treated by intragastric administration of sterilized physiological sodium chloride solution. Drug group rats were exposed to CS as described in the CS group above, to induce respiration deficiency syndrome associated with lung. They were simultaneously treated with EtOAcF at does of 400 mg/kg by intragastric administration for 15 days. Positive group as described in the drug group above instead of Golong brand Kechuanning capsules at a dose of 1.2 g/kg.

### 3.5. Antitussive effects

Antitussive assays were carried out as previously reported [13,14]. Unrestrained conscious guinea pigs were individually placed in a transparent 20 cm × 10 cm × 10 cm airtight Plexiglass chamber and exposed to 17.5% (w/v) citric acid aerosol produced by an ultrasonic nebulizer for 1 min with a flow rate of 0.6 mL/min, the aerosol particles had a mass median aerodynamic diameter of 0.9 μm. During the 10 min observation period, the guinea pigs were continuously observed, and the latent period of cough and numbers of coughs were simultaneously noted. The respiratory wave patterns were recorded by a pressure transducer connected to the MedLab [15]. Guinea pigs coughing more than 18 times but less than 30 in the first challenge were selected for further antitussive tests. 

### 3.6. Pulmonary function

The effect of the EtOAcF on lung function changes in rats with respiration deficiency syndrome associated with lung was observed by using MedLab. Rats were anesthetized with entobarbital sodium (30 mg/kg) and placed in a whole body plethysmograph, then *R_L_* and *C_dyn_* were monitored for 30 min and maximal changes from baseline for each parameter were recorded. The effect of EtOAcF was determined by comparing the CS-induced changes in *R_L_* and *C_dvn_* after drug treatment with the mean of responses alone in the same rat on previous and successive control periods.

### 3.7. Cytokine concentrations in plasma by ELISA

The concentrations of IL-8, IL-10, ET-1, GM-CSF and TNF-α in serum were determined using enzyme linked immunosorbent assay (ELISA) kit, according to the manufacturer’s instructions (R&D Systems, Inc., USA).

### 3.8. Statistical analysis

Data were evaluated using the T-test followed by a least significant difference test using SPSS/11.5 software. P values of less than 0.05 were considered to indicate a significant difference between treatments. All values are expressed as mean ± S.E.M. 

## 4. Conclusions

TZS, commonly known as a type of “qi-enriching and tonic drug”, is widely used in traditional Chinese medicine to treat “respiration deficiency syndrome associated with lung” diseases similar to COPD in the stable phase. However, its mechanism of action is not clear.

Ethanol, water and ethyl acetate extracts of TZS were found to be effective cough suppressors. The EtOAcF was found to be the most active when compared to the others at the same dose and exhibited a dose-dependent antitussive effect between 100 to 500 mg/kg. EtOAcF protects lung against an increase of *R_L_* and reduction of *C_dvn_* induced in the stable phase COPD rat model. EtOAcF treatment significantly decreased the expression of IL-8, ET-1 and TNF-α in serum and also markedly decreased the level of granulocyte macrophage-colony stimulating factor (GM-CSF). The anti-inflammatory cytokine, IL-10, slightly increased. 

GM-CSF is a big name for a tiny protein made by the body which acts to perpetuate an ongoing inflammatory response within the lungs, one of the key problems in COPD. New findings suggest that blocking this little protein can reduce or prevent smoking-related inflammation in mice. Cytokines are distinct proteins which are primarily produced by white blood cells and act as messengers between our cells. GM-CSF is just one of these types of cytokines. Cigarette smoke triggers the release of GM-CSF and other cytokines which activate and recruit inflammatory cells inside our lungs, eventually destroying lung tissue, causing emphysema [23,24,25,26]. It is noteworthy that TZS suppressed GM-CSF from the epithelium and dramatically inhibited the production of IL-8 in combination with TNF-α. IL-10 inhibits the release of pro-inflammatory cytokines and other mediators may counteract the inflammatory process in order to re-establish homeostasis. TZS was found to non-significantly increased levels of IL-10. The results of our study have demonstrated an improvement in the ratio of IL-10/TNF-α that has risen from 21.9 percent to 38.4 percent. This was due not only to an accentuated increase in IL-10 but also to a reduction in the TNF-α level. Thus, the balance of pro- (TNF-α) and anti- (IL-10) inflammatory cytokines may favor a more anti-inflammatory milieu, confirmed by the increased IL-10 to TNF-α ratio after TZS administration [27,28]. 

TZS may have a potential beneficial effect, based upon the strong synergistic action, to prevent or alleviate the clinical symptoms of COPD, particularly in moderate and mild cases. It is likely that components derived from TZS contained in the EtOAcF improved pulmonary function via inhibition of airway inflammation by adjustment of multi-cytokine levels.
